# A Comparative Study of Harmonic Scalpel Versus Suture Ligation for Appendix Base Closure in Patients With Appendicitis Undergoing Laparoscopic Appendectomy: A Randomized Controlled Trial

**DOI:** 10.7759/cureus.88560

**Published:** 2025-07-22

**Authors:** Apoorva Mathur, Gulab Dhar Yadav, Shraddha Verma, Priyesh Shukla

**Affiliations:** 1 Department of General Surgery, Ganesh Shankar Vidhyarthi Memorial Medical College, Kanpur, IND

**Keywords:** appendiceal stump closure, appendicitis, harmonic scalpel, laparoscopic appendectomy, operative time, surgical site infection, suture ligation

## Abstract

Background and objective

Appendicitis is a common cause of acute abdominal pain and a frequent indication for emergency abdominal surgery. Laparoscopic appendectomy has become the preferred surgical treatment due to its many advantages over open appendectomy, including reduced morbidity, shorter hospital stay, and faster recovery. A critical step in laparoscopic appendectomy is the secure closure of the appendix stump to prevent postoperative complications. While laparoscopic appendectomy is widely practiced, there is no consensus on the optimal method for appendix stump closure. This prospective study aimed to compare the harmonic scalpel (HS) with suture ligation (SL) for appendix base closure in terms of operative time, surgical site infection (SSI), postoperative pain, postoperative ileus, and hospital stay.

Methods

This prospective randomized controlled trial was conducted at the Department of General Surgery, GSVM Medical College, Kanpur, involving 60 patients with appendicitis undergoing laparoscopic appendectomy. The data entry was performed using Microsoft Excel. The final statistical analysis was carried out using SPSS Statistics version 25.0 (IBM Corp., Armonk, NY). Continuous variables were presented as mean ± standard deviation (SD) or median with interquartile range (IQR), depending on data distribution. Categorical variables were expressed as frequencies and percentages. A p-value <0.05 was considered statistically significant.

Results

A total of 60 patients were randomized into two groups (30 each): HS and SL. The most affected age group was 18-42 years, with a male-to-female ratio of 1.2:1. The mean operative time was significantly shorter in the HS group (28.4 ± 6.2 minutes) compared to the SL group (43.3 ± 7.1 minutes, p<0.05). Postoperative pain scores were lower in the HS group at six, 24, and 48 hours (p<0.05). SSI (6.6% vs. 13.3%), postoperative ileus (6.6% vs. 13.3%), and readmission rates (3.3% vs. 10%) were all lower in the HS group. Hospital stay was also reduced (2.8 ± 1.2 vs. 3.5 ± 1.4 days, p<0.05), and patients returned to normal activities earlier.

Conclusions

This study demonstrates HS is a safe and effective alternative to suture ligation, offering improved surgical outcomes and faster recovery for appendiceal stump closure during laparoscopic appendectomy. HS significantly reduces operative time, postoperative pain, complication rates, hospital stay, and readmissions, while enhancing recovery and overall patient outcomes.

## Introduction

Appendicitis is one of the most prevalent acute abdominal conditions requiring emergency surgical intervention. The shift from open to laparoscopic appendectomy has been driven by the benefits of its minimally invasive nature, including reduced postoperative pain, faster recovery, and improved cosmetic outcomes [1,2]. A critical component of laparoscopic appendectomy is the secure closure of the appendiceal stump, as inadequate closure can lead to serious complications such as intra-abdominal abscess, peritonitis, and fistula formation. Several techniques for stump closure have been developed, including endo-loops, endoscopic staplers, titanium and polymer clips, intracorporeal sutures, and advanced energy devices like bipolar cautery, LigaSure, and the harmonic scalpel (HS) [3]. Despite their widespread use, there is no consensus on the optimal method, particularly in resource-limited settings where cost-effectiveness and simplicity are key considerations [4].

Suture ligation (SL) remains a cost-effective and widely used method in laparoscopic appendectomy. It does not require expensive surgical instruments and can be performed in low-resource settings. However, it is technically demanding, requiring advanced laparoscopic knot-tying skills, and is associated with longer operative times compared to other techniques. Additionally, if the knot is not tied securely, there is a risk of stump leak, leading to potential postoperative complications [5]. HS is an ultrasonic energy-based device that enables simultaneous tissue sealing and cutting, offering a highly efficient alternative to SL. The device operates by converting ultrasound energy into mechanical energy at the active blade. Unlike electrocautery, which uses high temperatures to burn tissue, HS operates at a lower temperature (50-100 °C), reducing thermal injury to surrounding tissues [6,7]. The device divides tissue by using high-frequency (55,000 Hz) ultrasonic energy transmitted between the instrument blades. The active blade of the instrument vibrates longitudinally against an inactive blade over an excursion of 50-100 μm.

Despite its many benefits, HS has certain limitations. It is expensive, making it less accessible in resource-limited settings. Additionally, its use requires specialized training, and there is a learning curve for surgeons to effectively utilize the device. The availability of the device is another concern, as it may not be present in all surgical centers [8]. This study aimed to compare HS and SL techniques for base closure of the appendix during laparoscopic appendectomy, focusing on operative time, postoperative complications, pain, and length of hospital stay. The findings may help identify a safer, more efficient, and economically viable method for stump closure in laparoscopic appendectomy.

## Materials and methods

Study design

This prospective randomized controlled trial was conducted in the Department of General Surgery at GSVM Medical College, Kanpur. It spanned a period of one year from April 2024 to April 2025 and included patients with appendicitis undergoing laparoscopic appendectomy. This study was approved by the Ethics Committee (For Biomedical Health & Research), GSVM Medical College, Kanpur, under Protocol no. EC/BMHR/2024/35. This study was registered on ClinicalTrial.gov with the identifier NCT07049965.

Study sample

Sixty patients with clinically and radiologically confirmed acute appendicitis were randomized into two groups: Group HS and Group SL, each comprising 30 patients. The inclusion and exclusion criteria are summarized in Table 1. Patients presenting to the outpatient department (OPD) who met the inclusion criteria were screened and enrolled. Eligible participants were then randomly allocated into two groups using a computer-generated randomization list. Allocation was concealed using sealed, opaque envelopes to prevent selection bias. Before recruitment, all patients were informed about potential risks, and informed consent was obtained regarding their willingness to participate.

**Table 1:** Inclusion and exclusion criteria of the study CAD: coronary artery disease; CHF: congestive heart failure; COPD: chronic obstructive pulmonary disease

Inclusion criteria
Age between 5–65 years
Any gender
Patient willing to undergo laparoscopic appendectomy
Acute appendicitis confirmed clinically and radiologically
Alvarado score ≥7
Alvarado score ≥5 with positive ultrasound findings

Exclusion criteria
The base of the appendix is highly inflamed or gangrenous
Presence of any intra-abdominal pathology other than acute appendicitis, such as:
Appendicular mass
Appendicular abscess
Tuberculosis
Carcinoid tumor
Pelvic inflammatory disease
Ovarian tumors
Caecal mass
Patients declared unfit for laparoscopic surgery during preoperative assessment
Patients with severe comorbid conditions (e.g., COPD, CAD, CHF)

Patients with a gangrenous appendix or a severely inflamed appendix base identified intraoperatively were excluded from the study. This determination was made by the operating surgeon based on direct visual assessment during laparoscopy, before the application of the closure technique. We acknowledge that this constitutes a post-randomization exclusion, which may introduce potential selection bias. However, this step was necessary to maintain procedural safety and ensure a valid comparison between HS and SL techniques.

Diagnosis

Patients having a score of 7 or greater as per the Alvarado scoring system were deemed to have a confirmed diagnosis of acute appendicitis, and those patients who had a score of 5 or 6 were suspected to have acute appendicitis (Table 2). All patients with a score of 5 or greater underwent urgent ultrasound to either confirm the diagnosis or rule out complications. Ultrasound findings suggestive of acute appendicitis were aperistaltic, non-compressible, dilated appendix; presence of appendicolith; distinct appendiceal wall layers; echogenic prominent pericaecal fat; periappendiceal fluid collection; and target appearance (axial section).

**Table 2:** Alvarado scoring system The Alvarado score was used for the clinical diagnosis of acute appendicitis [9]. This scoring system is in the public domain and does not require permission for academic use RIF: right iliac fossa

Alvarado scoring system - symptoms and signs	Score
Symptoms	
Migratory RIF pain	1
Anorexia	1
Nausea and vomiting	1
Signs	
Tenderness	2
Rebound tenderness	1
Elevated temperature	1
Laboratory findings	
Leucocytosis	2
Shift to the left (segmented neutrophils)	1

Methodology

All surgeries were performed under general anesthesia by consultant surgeons following standard aseptic precautions. Patients undergoing laparoscopic appendectomy with either HS or SL for appendix base closure were enrolled in a 1:1 ratio and allocated to Group HS or Group SL. In Group HS, HS was used for both sealing and cutting the appendix base. In Group SL, the base was secured with intracorporeal ligatures with Polyglactin (Vicryl) 1-0 absorbable sutures before division. All other operative steps, including port placement and mesoappendix management, were standardized across both groups [9]

**Figure 1:**
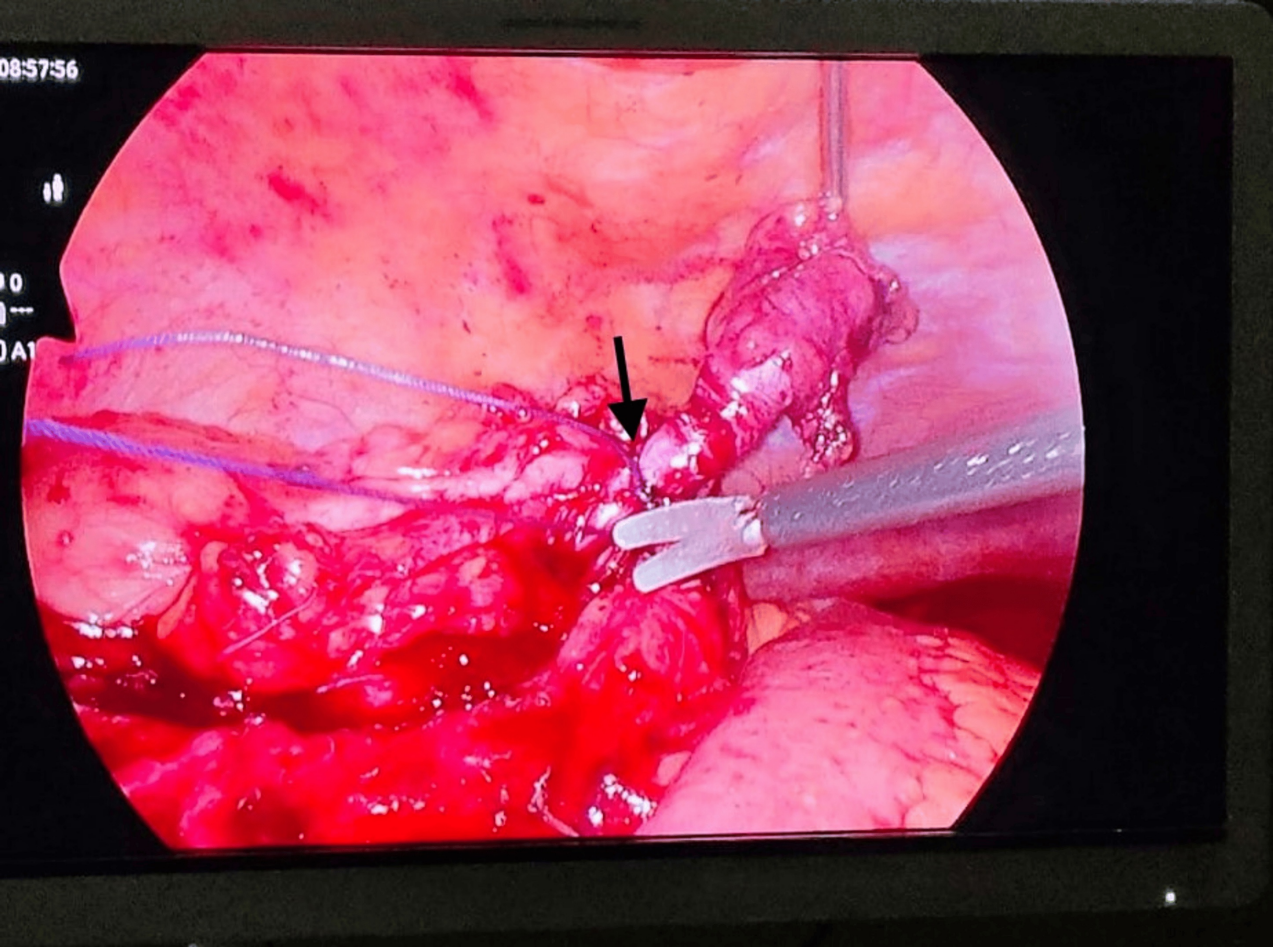
Laparoscopic appendectomy using suture ligation The arrow indicates the suture ligature applied at the base of the appendix

**Figure 2:**
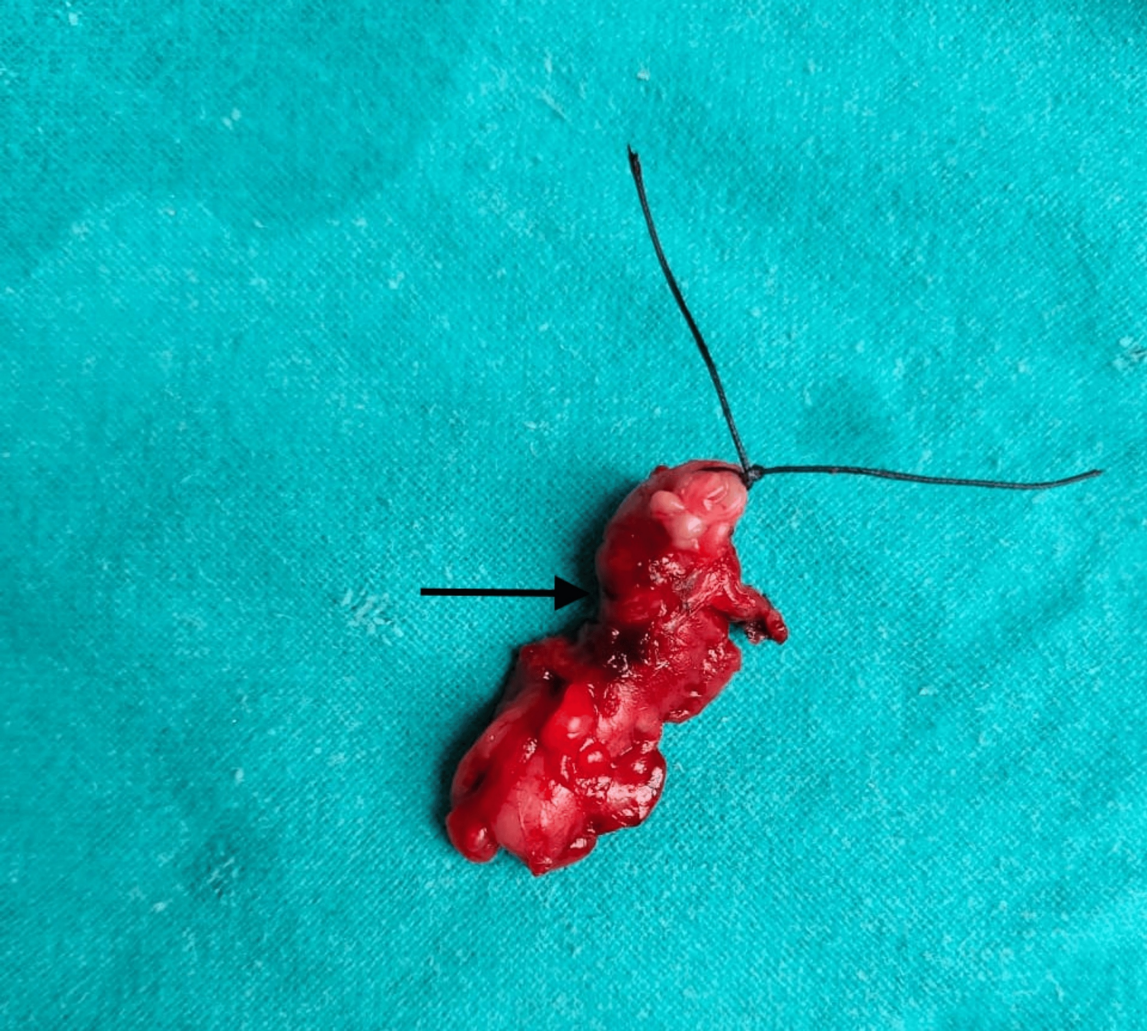
Specimen of appendix after laparoscopic appendectomy using suture ligation The arrow points to the appendix specimen

Intraoperative findings, including the position of the appendix and the duration of surgery, were documented. Photographic documentation was obtained during surgery. Laparoscopic appendectomy using the SL technique is demonstrated in Figure 1, with the resected specimen shown in Figure 2.

**Figure 3:**
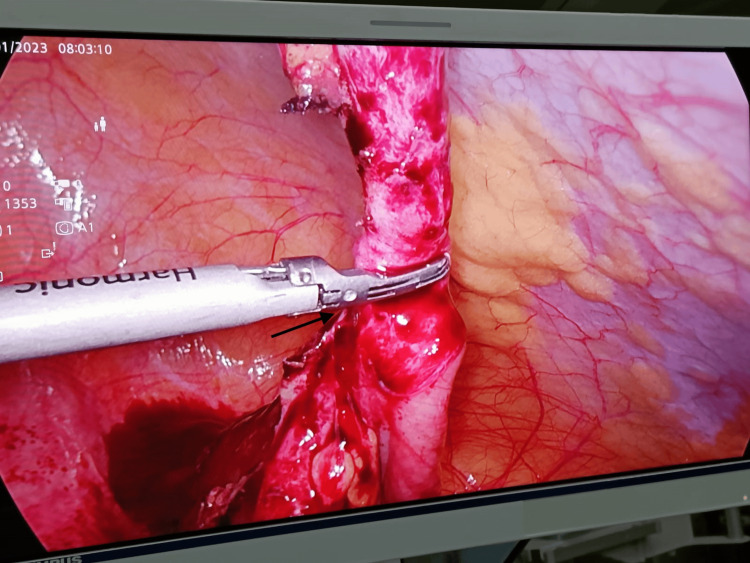
Laparoscopic appendectomy using harmonic scalpel The arrow indicates the harmonic scalpel being used to seal and divide the base of the appendix

**Figure 4:**
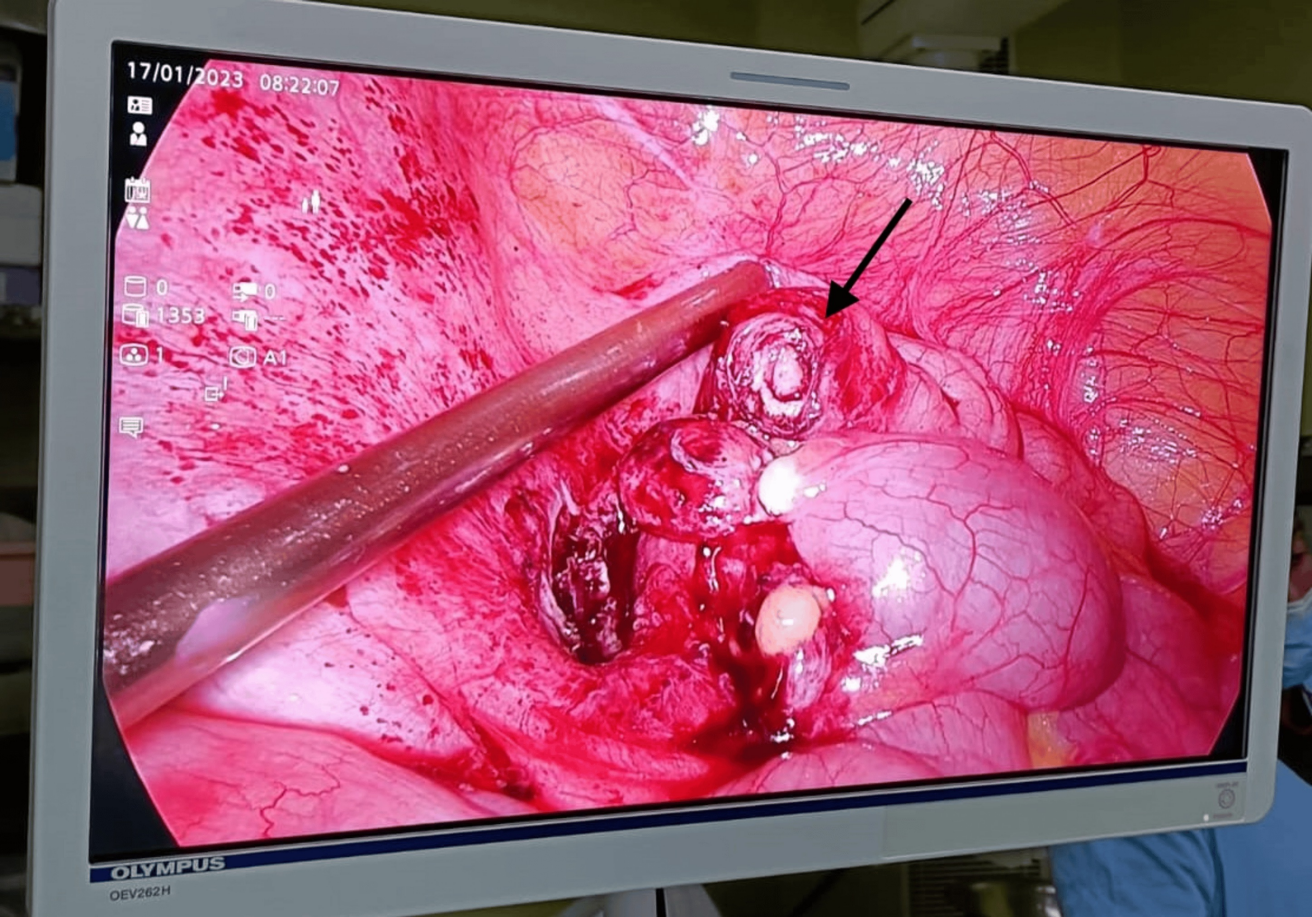
Laparoscopic view highlighting the appendix stump following appendectomy using harmonic scalpel The arrow indicates the precisely sealed and transected base

**Figure 5:**
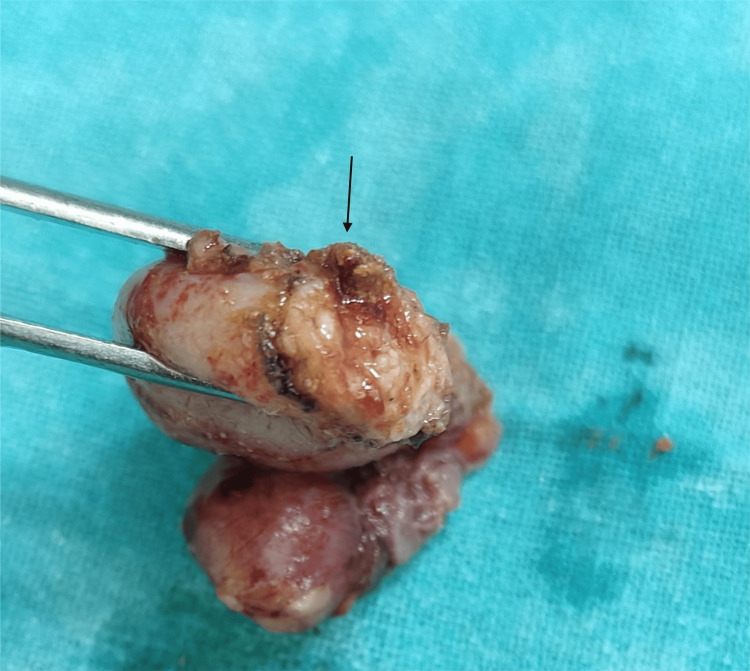
Specimen of appendix after laparoscopic appendectomy using harmonic scalpel for seal and cut The arrow shows the sealed and divided base of the appendix

The use of HS for sealing and transecting the appendix base is illustrated in Figures 3, 4, and the corresponding specimen is shown in Figure 5. 

Outcome assessment

Outcomes assessed included operative time, postoperative pain (visual analog scale (VAS), postoperative leak, localized peritonitis, postoperative ileus, surgical site infections (SSI), hospital stay, and return to regular diet and activities. The outcomes of the study were categorized into primary and secondary endpoints. The primary outcome of the study was operative time, selected to assess the intraoperative efficiency of the appendix base closure technique used in each group. The secondary outcomes included postoperative pain, which was assessed using VAS at six, 12, and 24 hours post-surgery; length of hospital stay (in days); time to return to oral intake; time to resume normal daily activities; postoperative complications such as postoperative leakage, postoperative ileus, SSI; and readmission within 30 days. The intraoperative time (in minutes) was measured from the skin incision until the last skin suture was applied for closure. Postoperative pain was evaluated using VAS at six, 24, and 48 hours postoperatively. The number of nights spent in the hospital was used to determine the length of hospital stay.

The first intravenous painkiller injection was given immediately post-surgery. The second and third doses were administered 12 and 24 hours later. Further doses were given depending on the adequacy of pain control. Postoperative pain was assessed using VAS by a blinded independent observer who was not involved in the surgical procedure or patient group allocation. Postoperative ileus was defined as the failure to pass flatus or stool accompanied by absent bowel sounds for more than 24 hours after surgery. SSIs were identified based on clinical signs, including erythema, tenderness, purulent discharge, or fever, and were assessed both during the hospital stay and at the first outpatient follow-up, which typically occurred within 7-10 days postoperatively.

Return to normal activities was defined as the number of days from surgery until the patient reported resuming their usual daily routine (e.g., household tasks, work, or school) without significant limitation. This information was recorded during scheduled postoperative follow-up visits (at one week, one month, and three months), based on patient self-report using a standardized recovery questionnaire.

Follow-up evaluations were conducted at one month and three months; patients were assessed for long-term complications, including the development of incisional hernia, persistent or chronic abdominal pain, and delayed SSI. Wound healing was evaluated, and patients were questioned regarding any recurrence of abdominal symptoms or functional limitations. Readmissions or additional interventions since discharge were also recorded.

Statistical analysis

The data entry was performed using Microsoft Excel, ensuring accuracy and organization of collected patient information. The final statistical analysis was carried out using SPSS Statistics version 25.0 (IBM Corp., Armonk, NY). Descriptive statistics were used to summarize the demographic data, clinical characteristics, and surgical outcomes. Continuous variables were presented as mean ± standard deviation (SD) or median with interquartile range (IQR), depending on data distribution. Categorical variables were expressed as frequencies and percentages. Comparative analysis between groups was conducted using independent t-tests or Mann-Whitney U tests for continuous variables and Chi-square or Fisher’s exact tests for categorical variables. A p-value <0.05 was considered statistically significant.

## Results

Demographics and clinical characteristics of the study population

The mean age in the HS group was 31.2 ± 13.3 years, while that in the SL group was 29.3 ± 11.2 years. The age distribution showed no significant difference between the two groups (p>0.05), ensuring comparability (Table 3). During surgery, the most common appendix position was retrocecal (65%), followed by pelvic (26.7%) and pre-ileal (8.3%). The distribution of appendix positions between the two groups was not significantly different, further supporting baseline comparability (Table 3).

**Table 3:** Baseline characteristics of the study population Gender distribution compared using the Chi-square test. P-value <0.05 was considered statistically significant HS: harmonic scalpel; SD: standard deviation; SL: suture ligation

Parameter	HS Group (n=30)	SL Group (n=30)	P-value
Age, years, mean ± SD	31.2 ± 13.3	29.3 ± 11.2	__
Gender, n (%)			
Male	18 (60%)	17 (56.7%)	0.785
Female	12 (40%)	13 (43.3%)	0.785
Male-to-female ratio	1.5:1	1.3:1	—
Appendix position, n (%)			
Retrocecal	19 (63.3%)	20 (66.7%)	—
Pelvic	9 (30%)	7 (23.3%)	—
Pre-ileal	2 (6.7%)	3 (10%)	—

Distribution of patients based on Alvarado score

**Figure 6:**
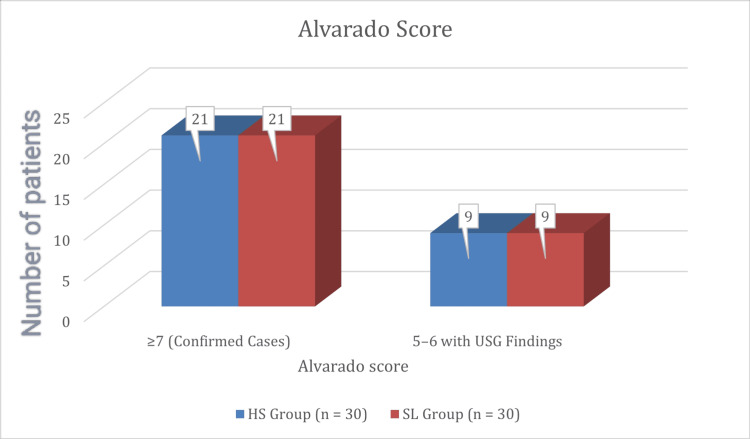
Distribution based on Alvarado score HS: harmonic scalpel; SL: suture ligation; USG: ultrasound sonography

The Alvarado score is a widely accepted diagnostic criterion for acute appendicitis. In this study, patients were classified based on their Alvarado scores, ensuring uniformity between the two groups. Both groups had comparable distributions of Alvarado scores, as illustrated in Figure 6.

Operative time

A major component of this study involved comparing the operative time between the two techniques. The HS group had a significantly shorter operative time (28.4 ± 6.2 minutes) compared to the SL group (43.3 ± 7.1 minutes) (p<0.05). These findings suggest that HS significantly reduces operative time, making it a more efficient technique than suture ligation (Table 4).

**Table 4:** Operative time comparison Data analyzed using an independent t-test. P-value <0.05 was considered statistically significant HS: harmonic scalpel; SD: standard deviation; SL: suture ligation

Group	Operative time, minutes, mean ± SD	P-value
HS Group	28.4 ± 6.2	<0.05
SL Group	43.3 ± 7.1	—

Postoperative complications

The occurrence of postoperative ileus was lower in the HS group (6.6%) compared to the SL group (13.3%), but this difference was not statistically significant (p>0.05). SSIs were observed in both groups, with a numerically lower frequency in the HS group (6.6% vs. 13.3%); however, this difference also did not reach statistical significance (p>0.05) (Table 5). One of the key concerns following appendectomy is the risk of postoperative leakage, localized peritonitis, or pus collection. These complications occurred more frequently in the SL group, yet none of the differences were statistically significant (p>0.05), as summarized in Table 5.

**Table 5:** Postoperative complications Data analyzed using Fisher's exact test. P-value <0.05 was considered statistically significant HS: harmonic scalpel; SL: suture ligation

Postoperative complication	HS Group (n=30), n (%)	SL Group (n=30), n (%)	P-value
Postoperative ileus	2 (6.6%)	4 (13.3%)	>0.05
Surgical site infection	2 (6.6%)	4 (13.3%)	>0.05
Postoperative leak	1 (3.3%)	2 (6.6%)	>0.05
Localized peritonitis	1 (3.3%)	2 (6.6%)	>0.05
Pus collection	0 (0%)	1 (3.3%)	>0.05

Postoperative pain and recovery

To quantify postoperative pain, patients' VAS scores were recorded at six, 24, and 48 hours postoperatively. Patients in the HS group experienced significantly lower postoperative pain at all time intervals, as shown in Table 6.

**Table 6:** Pain score (VAS scale) at 6, 24, and 48 hours post-surgery Data analyzed using an independent t-test. P-value <0.05 was considered statistically significant HS: harmonic scalpel; SL: suture ligation; VAS: visual analog scale

Time interval	HS Group (mean VAS score ± SD)	SL Group (mean VAS score ± SD)	P-value
6 hours	3.2 ± 1.1	4.1 ± 1.3	<0.05
24 hours	2.43 ± 0.5	3.7 ± 0.59	<0.05
48 hours	2.1 ± 0.8	3.0 ± 1.1	<0.05

Hospital stay and recovery

The duration of hospital stay is an essential indicator of postoperative recovery and cost-effectiveness. Patients in the HS group had significantly shorter hospital stays compared to the SL group, indicating faster recovery times (Table 7). The time taken for patients to tolerate oral intake postoperatively is another key metric of recovery. Table 7 shows the time taken to resume a normal diet postoperatively in both groups. The HS group resumed normal oral intake significantly earlier than the SL group. A critical component of postoperative recovery is the ability to resume normal daily activities, including work or school. The HS group returned to daily activities significantly faster than the SL group (Table 7). This is a significant finding that reinforces the enhanced recovery profile of the HS technique

**Table 7:** Postoperative recovery outcomes Data analyzed using an independent t-test. P-value <0.05 was considered statistically significant HS: harmonic scalpel; SD: standard deviation; SL: suture ligation

Parameter	HS Group, mean ± SD	SL Group, mean ± SD	P-value
Hospital stay, days	2.8 ± 1.2	3.5 ± 1.4	<0.05
Time to resume oral diet, hours	24.5 ± 4.2	38.2 ± 5.7	<0.05
Time to resume normal activities, days	4.8 ± 1.3	7.3 ± 2.1	<0.05

Readmission rates within 30 days post-surgery

The 30-day readmission rate was evaluated as a key indicator of postoperative safety (Table 8). The SL group had a higher readmission rate, although this difference did not reach statistical significance (p>0.05).

**Table 8:** Readmission rates Data analyzed by Fisher's exact test. P-value <0.05 was considered statistically significant HS: harmonic scalpel; SL: suture ligation

Group	Readmissions, n (%)	P-value
HS Group	1 (3.3%)	>0.05
SL Group	3 (10%)	—

Follow-up data at one month and three months

Postoperative follow-up at one month and three months was conducted to assess residual symptoms, recurrence, or long-term complications, as summarized in Table 9. During follow-up visits, patients were assessed for residual or recurrent abdominal pain, nausea, vomiting, bowel irregularities, fever, and signs of wound-related complications such as discharge or redness. Assessment was based on both clinical examination and patient self-report. Long-term complications were assessed during the three-month follow-up period and included the presence of incisional hernia, adhesive intestinal obstruction, or chronic abdominal pain. At the end of the three-month follow-up, 100% (30/30) of patients in the HS group and 93.3% (28/30) in the SL group were symptom-free.

**Table 9:** Follow-up data at one month and three months Data analyzed using the Chi-square test. P-value <0.05 was considered statistically significant HS: harmonic scalpel; SL: suture ligation

Follow-up period	HS Group, n (%)	SL Group, n (%)	P-value
No symptoms (1 month)	29 (96.7%)	27 (90%)	>0.05
No symptoms (3 months)	30 (100%)	28 (93.3%)	>0.05
Long-term complications	0 (0%)	0 (0%)	>0.05

## Discussion

This prospective study aimed to evaluate the efficacy of HS compared to SL for appendiceal stump closure during laparoscopic appendectomy. This study contributes to the growing body of literature by comparing HS and SL across multiple parameters, including operative efficiency, postoperative pain, complication rates, hospital stay, and overall recovery. The findings suggest that HS offers significant advantages over SL, supporting its broader adoption in clinical practice.

Demographics

The highest incidence of appendicitis was observed in males aged 18-42 years, as shown in Table 3. This distribution aligns with previous findings, including those reported by Körner et al. [10], who also demonstrated a higher incidence in males within this age group.

Operative time and surgical efficiency

A key finding of this study was the significantly reduced operative time associated with HS. The mean operative duration was 28.4 ± 6.2 minutes in the HS group compared to 43.3 ± 7.1 minutes in the SL group (p<0.05) (Table 4). This is in line with previous reports by Borkar et al. [11] and Pogorelić et al. [12], who demonstrated that energy-based devices notably decrease operative time. The efficiency of HS can be attributed to its multifunctionality, sealing and cutting tissue simultaneously, thus eliminating the need for frequent instrument exchanges. In contrast, SL requires multiple steps, including knot-tying, which are technically demanding in a laparoscopic environment and can prolong the procedure. Additionally, HS is associated with minimal lateral thermal spread, enhancing safety in the confined pelvic space [13,14].

Postoperative pain and analgesia requirements

Postoperative pain management plays a critical role in recovery. In this study, pain scores measured using VAS were significantly lower in the HS group at six, 24, and 48 hours postoperatively (p<0.05) (Table 6). These findings are supported by prior research [13], suggesting that the reduced postoperative pain associated with HS is likely due to lower tissue trauma and decreased inflammatory response. Unlike SL, which involves the placement of foreign material (sutures) that may provoke local irritation and inflammation, HS offers a cleaner dissection and reduced nociceptive activation [12]. From a clinical standpoint, better pain control facilitates early ambulation, reduces opioid consumption, and decreases the risk of complications such as venous thromboembolism and pulmonary issues.

Postoperative complications and surgical site infections

The incidence of SSIs was lower in the HS group (6.6%) compared to the SL group (13.3%) (Table 5), consistent with findings from previous studies [13]. Although this difference was not statistically significant, the reduced SSI rate in the HS group may be attributed to its precise sealing capability, which minimizes tissue necrosis and bacterial colonization, which are key contributors to postoperative infection. In contrast, sutures used in the SL technique may act as a nidus for bacterial adherence. Postoperative ileus was also observed less frequently in the HS group (6.6%) than in the SL group (13.3%) (Table 5). While this difference did not reach statistical significance, it may reflect the less traumatic nature of dissection with the HS, resulting in reduced peritoneal irritation and cytokine-mediated inflammatory responses.

Hospital stay and recovery

Patients in the HS group had shorter hospital stays (2.8 ± 1.2 days vs. 3.5 ± 1.4 days; p<0.05) (Table 7) and resumed normal activities approximately two to three days earlier than those in the SL group (Table 7). These findings are in agreement with those of Raza et al. [15], who also reported faster recovery with the use of energy-based devices. The earlier discharge and return to daily activities likely stem from reduced pain, fewer complications, and minimized inflammatory responses. From a healthcare economics perspective, this translates to lower overall costs and resource utilization [16]. For patients, earlier recovery supports a quicker return to work or school, enhancing quality of life and reducing the socioeconomic burden.

Limitations

This study was conducted at a single center with a relatively small sample size and a short follow-up duration. The sample size was determined based on feasibility, time constraints, and the availability of eligible patients. Nonetheless, it is comparable to similar studies, including those by Khalid et al. and Hamdy et al. Another key limitation was the intraoperative exclusion of patients with a gangrenous or severely inflamed appendix base. Although these exclusions occurred after randomization, potentially introducing selection bias, they were clinically justified to ensure patient safety and maintain the integrity of the surgical procedure.

Future directions

Further multicenter trials, long-term follow-up studies, and comprehensive cost-benefit analyses are warranted to more strongly endorse the adoption of HS as the standard of care. Additionally, developing structured training programs to facilitate the transition from SL to HS may help standardize and optimize outcomes across varied surgical settings.

## Conclusions

HS appears to be a safe, effective, and efficient alternative to SL for appendiceal stump closure in selected patients undergoing laparoscopic appendectomy, within the limitations of our study. It significantly reduces operative time, postoperative pain, complications, and hospital stay. These advantages position HS as a favorable technique. With further validation through larger trials involving more diverse patient groups, it may be considered a preferred technique in appropriate clinical settings.
